# Small Cell Lung Cancer Transformation as a Resistance Mechanism to Osimertinib in Epidermal Growth Factor Receptor-Mutated Lung Adenocarcinoma: Case Report and Literature Review

**DOI:** 10.3389/fonc.2021.642190

**Published:** 2021-04-26

**Authors:** Alessandro Leonetti, Roberta Minari, Giulia Mazzaschi, Letizia Gnetti, Silvia La Monica, Roberta Alfieri, Nicoletta Campanini, Michela Verzè, Andrea Olivani, Luigi Ventura, Marcello Tiseo

**Affiliations:** ^1^Medical Oncology Unit, University Hospital of Parma, Parma, Italy; ^2^Pathology Unit, Department of Medicine and Surgery, University Hospital of Parma, Parma, Italy; ^3^Department of Medicine and Surgery, University of Parma, Parma, Italy; ^4^Unit of Infectious Diseases and Hepatology, University Hospital of Parma, Parma, Italy; ^5^Thoracic Surgery, Department of Medicine and Surgery, University of Parma, Parma, Italy

**Keywords:** NSCLC, EGFR, osimertinib resistance, SCLC transformation, phenotype switch

## Abstract

**Introduction:** Small cell lung cancer (SCLC) transformation represents a mechanism of resistance to osimertinib in *EGFR*-mutated lung adenocarcinoma, which dramatically impacts patients' prognosis due to high refractoriness to conventional treatments.

**Case Description:** We present the case of a patient who developed a SCLC phenotypic transformation as resistance mechanism to second-line osimertinib for T790M-positive *EGFR*-mutated NSCLC. Our patient received platinum–etoposide doublet following SCLC switch and achieved a modest clinical benefit which lasted 4 months. NGS and IHC analyses for p53 and Rb were performed on subsequent liver biopsies, revealing baseline *TP53* mutation and complete absence of p53 and Rb expression. Primary cell cultures were established following a liver biopsy at the time of SCLC transformation, and drug sensitivity assays showed meaningful cell growth inhibition when osimertinib was added to platinum–etoposide compared with control (*p* < 0.05). A review of the current literature regarding SCLC transformation after failure of osimertinib was performed.

**Conclusions:** Based on retrospective data available to date, platinum–etoposide chemotherapy is the preferred treatment choice in the occurrence of SCLC transformation after osimertinib failure. The extension of osimertinib in combination with chemotherapy in the occurrence of SCLC transformation as resistance mechanism to osimertinib is a matter of debate. The combination of osimertinib and platinum–etoposide was effective in inhibiting cell growth in our primary cell cultures. Clinical studies are needed to further explore this combination in the occurrence of SCLC transformation as a resistance mechanism to osimertinib.

## Introduction

The epidermal growth factor receptor (EGFR)-tyrosine kinase inhibitor (TKI) osimertinib constitutes a milestone for the treatment of advanced *EGFR*-mutated non-small cell lung cancer (NSCLC), both in the second line after failure of the previous generation of EGFR-TKIs due to the onset of T790M mutation and in the first line, regardless of T790M status (1). Despite remarkable activity exerted by osimertinib in this clinical setting, several resistance mechanisms have been described (2). Among these, small cell lung cancer (SCLC) phenotypic transformation represents a critical issue for clinicians, since effective therapeutic strategies to apply in this circumstance are lacking to date.

Herein, we report the case of a patient who developed a SCLC switch as resistance mechanism to second-line osimertinib for T790M-positive *EGFR*-mutated NSCLC, whose pre-clinical studies revealed a promising activity of prolonged osimertinib in combination with chemotherapy. Moreover, we performed a literature review to summarize the underlying mechanism and clinical features of SCLC transformation following osimertinib treatment, including current and future therapeutic opportunities.

## Case Description

**Figure 1:**
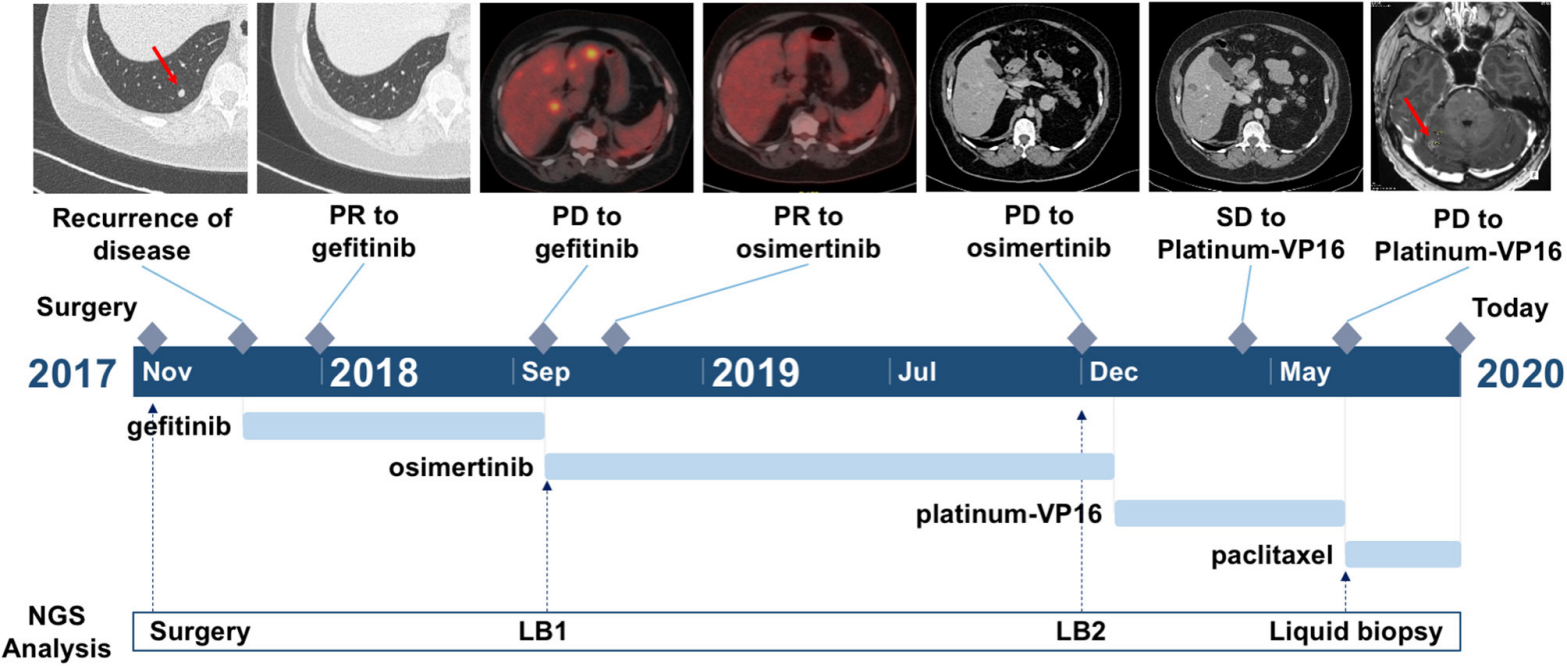
Timeline of the clinical course of the patient. Arrows indicate the link between the clinical event and the date of liquid biopsy time point.

**Figure 2:**
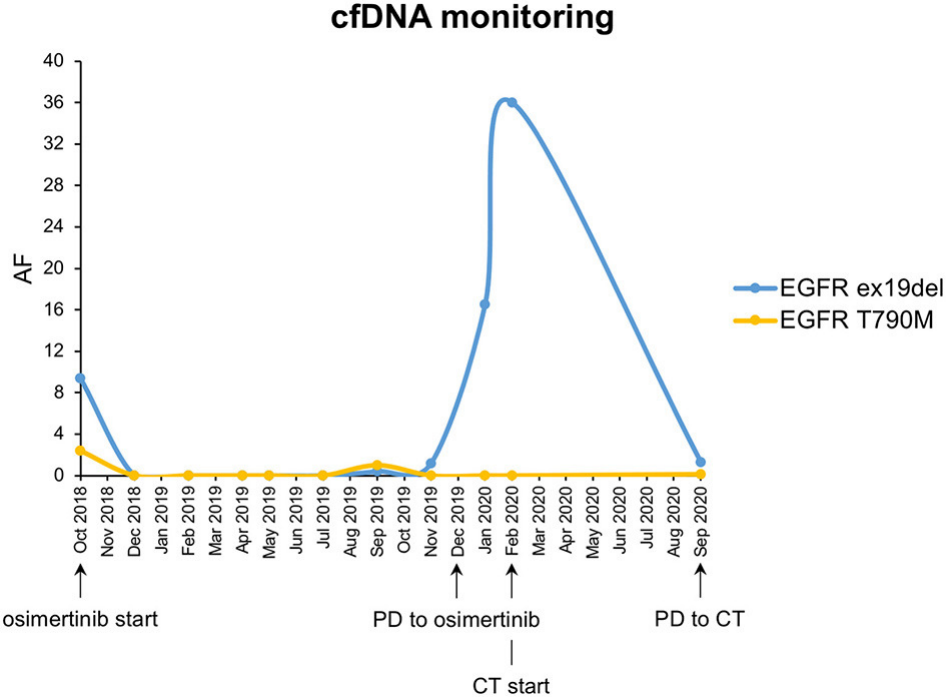
CfDNA monitoring of *EGFR* ex19del and *EGFR* T790M mutations. Each dot corresponds to a different liquid biopsy time point. AF, allele frequency; CT, chemotherapy with platinum–etoposide.

In September 2017, due to persistence of dry cough, a never-smoker 63-year-old woman underwent computed tomography (CT) scan which showed a lesion to the upper left pulmonary lobe associated with ipsilateral hilar lymph nodes. The subsequent positron emission tomography (PET) showed increased glucose uptake at both lesions. Left upper lobectomy and mediastinal lymphadenectomy were performed in November 2017, and the pathologic examination revealed an *EGFR*-mutated (exon 19 deletion) adenocarcinoma of the lung with stage pT3N2, R1 for microscopic residual disease at the bronchial margin. At the post-operative CT scan performed in January 2018, a recurrence of disease was documented, involving bilateral pulmonary metastases and left pleural effusion (Figure 1). Due to the presence of sensitizing *EGFR* mutation, the patient started gefitinib treatment, achieving partial response of the disease, with almost a complete disappearance of bilateral pulmonary nodules and a decrease of left pleural effusion. The benefit was maintained until October 2018, when the onset of multiple liver metastases and bone lesions was documented. A liver biopsy (liver biopsy 1, LB1) was subsequently performed in order to explore putative resistance mechanisms to gefitinib, revealing the presence of secondary T790M *EGFR* mutation in the context of exon 19 deletion. The presence of *EGFR* activating and T790M mutations was also confirmed on liquid biopsy (Figure 2). The patient promptly started osimertinib, which led to complete metabolic response on the liver and osteoblastic reaction of pre-existing bone lesions. Osimertinib therapy was continued until December 2019, when liver lesions increased. The patient underwent a liver biopsy on a new-onset lesion (LB2), which showed a phenotypic switch to SCLC.

Following LB2, primary cell line establishment was attempted. Tissue from liver metastasis was enzymatically digested using the Tumor Dissociation Kit (Miltenyi Biotec, Bergisch Gladbach, Germany), and the gentleMACS™ Dissociator (Miltenyi Biotec) was used for the mechanical dissociation in a closed and sterile system. The single-cell suspension was cultured in a 1:1 ratio of Ham's nutrient mixture F-12:DMEM, 10% FBS, 2 mM glutamine, 1× mammary epithelial growth supplement (MEGS, Life Technologies Corp., CA), and a Rho-associated protein kinase (ROCK) inhibitor.

**Figure 3:**
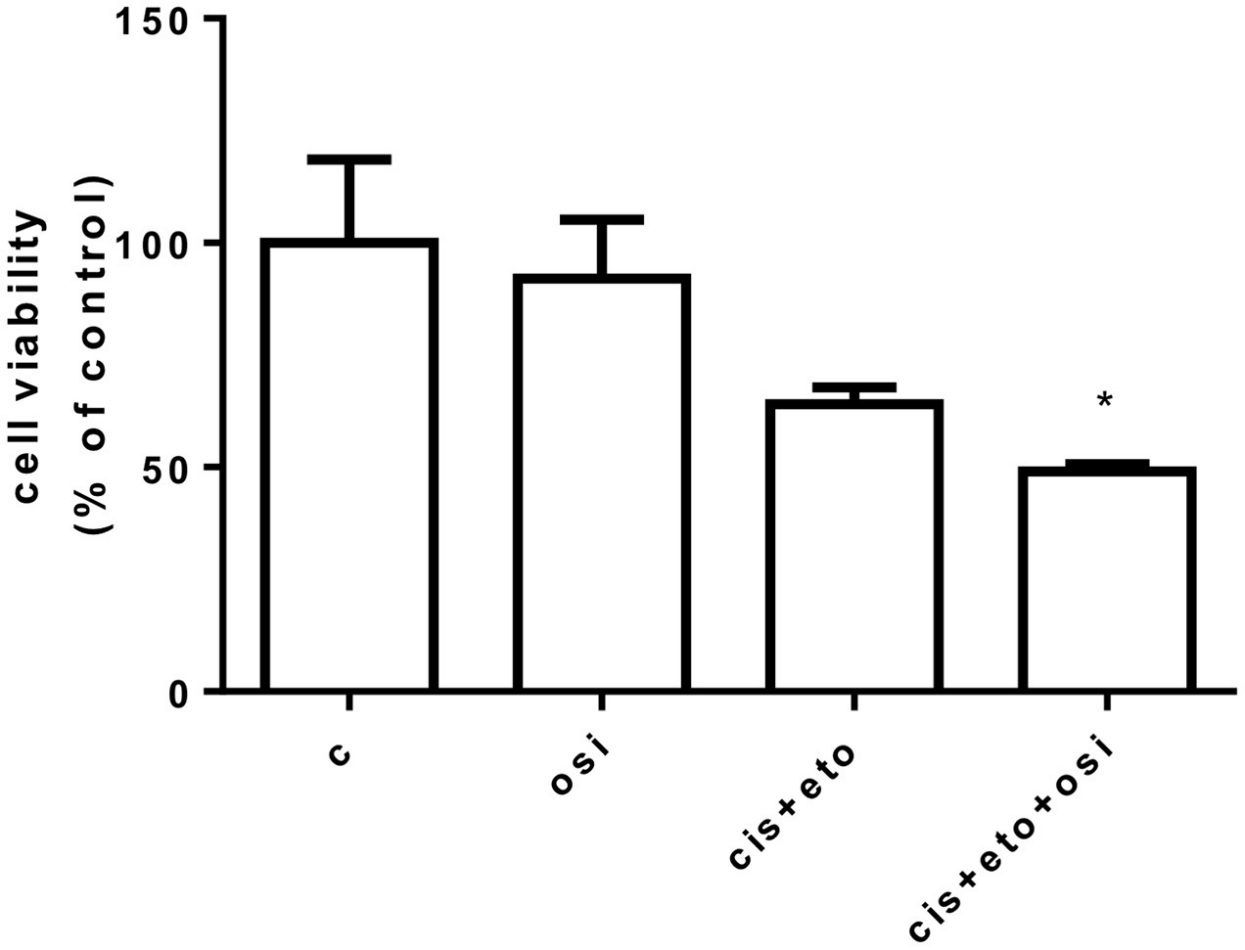
Drug screening in a primary cell line following osimertinib resistance. Primary cells were treated with 250 nM osimertinib, 0.150 μg/ml etoposide, and 0.05 μg/ml cisplatin. After 72 h, cell proliferation was assessed by CellTiter 96® AQueous One Solution Cell Proliferation Assay (MTS). Data are expressed as percent of cell viability vs. control cells and are means ± SD of three measurements. *p < 0.05.

A drug screening was performed in the primary cell culture testing osimertinib alone, cisplatin plus etoposide, and osimertinib combined with cisplatin plus etoposide. As shown in Figure 3, tumor cells were sensitive to the combination of osimertinib with chemotherapeutic agents (*p* < 0.05 vs. control) even if the results did not reach statistical significance vs. single drug treatments. Unfortunately, after a few weeks, the cells stopped their proliferation and it was not possible to perform additional experiments and to establish a stable cell line.

The patient underwent platinum–etoposide doublet in February 2020, and chemotherapy granted stability of the disease, as documented at the CT scan after three cycles. Unfortunately, following further three cycles, the patient experienced liver progression and central nervous system (CNS) progression due to the onset of multiple brain metastases (Figure 1). A further liver biopsy (LB3) was conducted on a new-onset liver lesion, with diagnosis of pure adenocarcinoma. At the time of writing the manuscript, the patient has been receiving weekly paclitaxel and whole-brain radiotherapy was performed. A liquid biopsy was carried out at the time of chemotherapy switch.

SCLC transformation clearly emerged as a mechanism of resistance to osimertinib. Nonetheless, next-generation sequencing (NGS) and immunohistochemistry (IHC) of Rb1 and p53 were performed in order to characterize the proficiency of the initial tumor to evolve in a neuroendocrine differentiated subtype. DNA was extracted from the liver biopsy undertaken before osimertinib treatment (LB1) and on SCLC-transformed liver lesion at osimertinib progression (LB2). Molecular analyses on LB3 were not conducted due to insufficient material. NGS study was performed with Solid Tumor Solution panel, Sophia Genetics, on MiSeq Platform, Illumina. No other putative resistance mechanisms to osimertinib were underlined (Table 1) and variants on *TP53* were found on LB1. The presence of those variants was retrospectively confirmed by NGS also in the lobectomy samples.

**Table 1:** NGS analyses on available samples.

	**Surgery (allelic** **frequency, %)**	**Pre-osimertinib**	**PD to osimertinib**	**PD to platinum–etoposide**
		**Tissue_LB1 (allelic frequency, %)**	**Tissue_LB2 (allelic frequency, %)**	**Liquid biopsy (allelic frequency, %)**
EGFR p.Glu746_Ala750del	49.60	64.50	45.30	1.30
EGFR T790M	–	18.40	–	0.13
TP53 p.Gln375^*^	50.60	66.70	38.10	0.47
TP53 p.His193Leu	25.4	31.80	19.70	–
BRAF p.Leu441Ile	–	5.70	–	–
NRAS p.Phe141Leu	–	5.40	–	–

The symbol “*” means an amino acid change in a stop codon (Ter, *) according to the Sequence Variant Nomenclature of Human Genome Variation Society.

**Figure 4:**
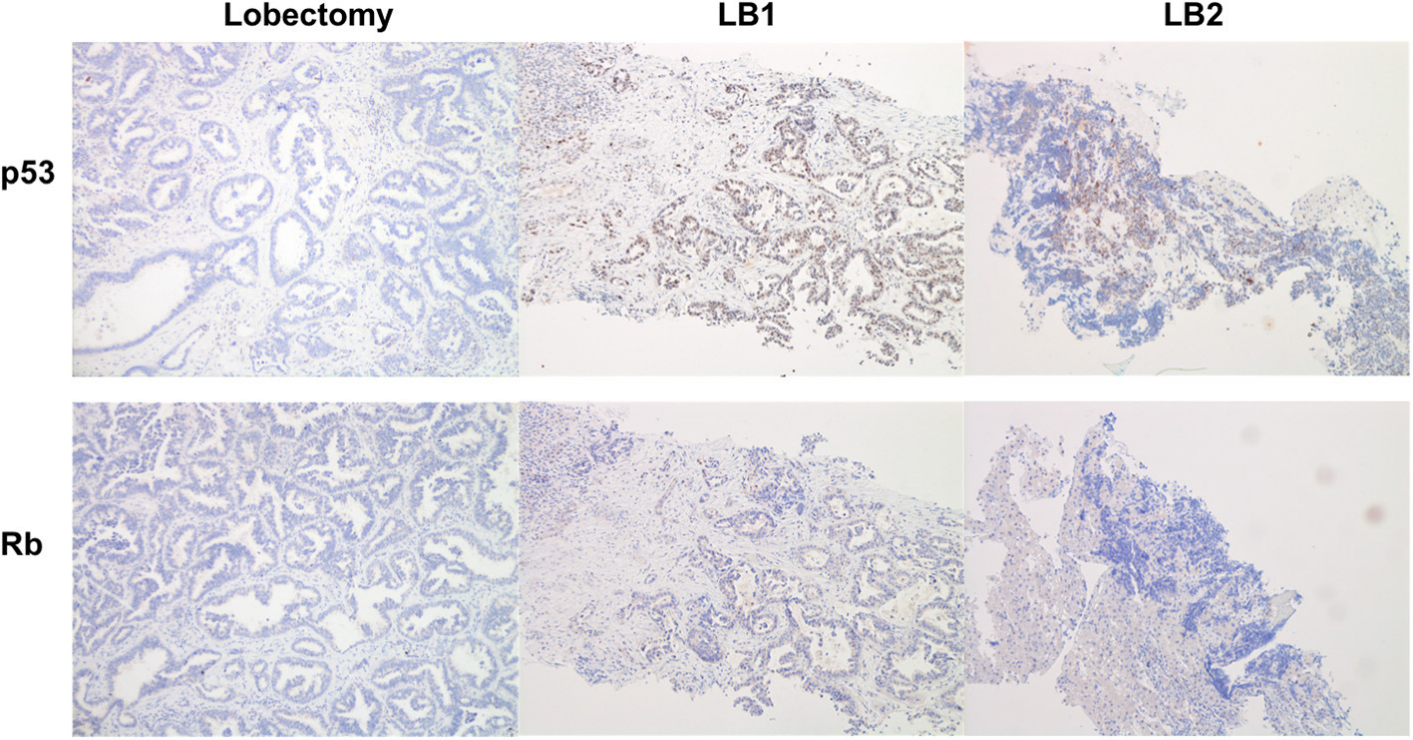
Immunohistochemistry analysis for p53 (upper line) and Rb (lower line). The resected tumor (lobectomy) showed a negativity for both p53 and Rb. In the first liver biopsy (LB1), p53 was overexpressed, whereas Rb was negative; the histotype of LB1 is that of a NSCLC/adenocarcinoma with morphology similar to lobectomy. In the second liver biopsy (LB2), we detected only scattered cells positive for p53 and negative for Rb; the histotype is that of a SCLC also documented by positivity for neuroendocrine markers.

Expression of p53 and Rb1 was evaluated with IHC on lobectomy tissue, LB1 and LB2, as previously described (3). IHC analysis was not performed on LB3 due to insufficient material. The evaluation of Rb1 showed the complete absence of expression in all analyzed samples, while p53 presented an abnormal pattern of expression consistent with inactivation. In fact, p53 was negative in the surgery tissue, overexpressed in LB1, and scattered positive in LB2 (Figure 4).

Moreover, NGS analysis was carried out on liquid biopsy collected after disease progression to third-line platinum–etoposide with AVENIO ctDNA Expanded Panel, Roche, on NextSeq Platform, Illumina. Interestingly, *EGFR* T790M showed up again with the known activating mutation and with two *TP53* non-sense variants (Table 1). The presence of *EGFR* mutations on liquid biopsy were confirmed also with ddPCR (Figure 2).

## Literature Review

### Incidence of SCLC Transformation in Osimertinib-Resistant Population

Dissecting the mechanisms of acquired resistance to osimertinib and other third-generation EGFR-TKIs represents an area of active investigation (2, 4, 5). Nonetheless, the role of histologic transformations, and more specifically SCLC switch, remains partly uncovered. This could be ascribed to the lack of analyses conducted on tissue samples; in fact, in both registrational trials, AURA and FLAURA, the delineation of genomic profiles of osimertinib-resistant NSCLC has been performed by NGS on plasma samples (6–8). Tissue biopsy at the time of progression to osimertinib plays a critical role in order to unravel SCLC transformation (9). Recent data from the six largest series of osimertinib-resistant cases to date reported an overall incidence of SCLC transformation ranging from 2 to 15% (10). As documented by Oxnard et al., among 28 patients who developed disease progression to second-line osimertinib and lost T790M, SCLC transformation resulted the most frequent mechanism of resistance accounting for 21% of all the recognized causes (11). Along the same line, Lee et al. reported small cell transformation as EGFR-independent resistance process in 5 over 36 tested cases (12). A lower proportion of SCLC-switched cases (4–6%) was present in “first-line” and “latter-line” cohorts included in the study by Schoenfeld et al. (13) and in osimertinib-resistant population described by Piotrowska et al. (14) and Michels et al. (15). In the real-world study conducted by Le et al., although potentially affected by the pure retrospective nature of the analysis, the incidence significantly decreased to 2% (16).

### Underlying Pathogenetic Mechanisms

Although histologic transformation is a well-known phenomenon, the comprehension of how it occurs and leads to EGFR-TKI resistance is still incomplete. SCLC transformation was first described in 2006 in a 45-year-old never-smoker woman with advanced *EGFR*-mutant adenocarcinoma after erlotinib failure (17). Since this initial observation, several additional cases have been reported (18), all confirmed by positive immunohistochemical staining for synaptophysin, chromogranin, or CD56/NCAM.

Different hypotheses have been proposed concerning the origin of SCLC as a mechanism of resistance to EGFR-TKIs. Initial studies on SCLC-transformed cancers have revealed relevant similarities to *de novo* SCLCs, most remarkably the inactivation of tumor suppression *via RB1* (19) and *TP53* (20). SCLC and NSCLC histologies might coexist within the same initial tumor, with the SCLC subtype becoming dominant after an initial response to EGFR-TKIs (18). Conversely, other lines of evidence support the assumption of a trans-differentiation process of the original *EGFR*-mutated adenocarcinoma under the pressure of TKIs (18, 21). Of interest, most of the SCLC-switched tumors retained the same *EGFR* mutation after transformation (22).

### Potential Predictors of SCLC Transformation and Therapeutic Options

The identification of biomolecular mediators of treatment-dependent SCLC transformation represents a fundamental goal to subsequently develop therapeutic interventions. Triple mutant adenocarcinomas (*EGFR/RB1/TP53*) are considered at higher risk for transformation to SCLC (23). Moreover, a rapid increase in the serum levels of neurone specific enolase (NSE) together with a poor response to EGFR-TKIs usually indicates a transformation from adenocarcinoma to SCLC (24). Along the same line, the assessment of the pro-gastrin-releasing peptide (pro-GRP) has also been recommended for the early prediction of disease transformation (25).

Since most of the SCLC-transformed cases harbor typical neuroendocrine differentiation, platinum–etoposide chemotherapy remains the current standard treatment at the time of SCLC switch (26). Even though SCLC-transformed cases achieve similar objective response rate from chemotherapy than primary SCLC (around 80%), the prognosis of the former is usually worse than the latter even after a favorable response (27). Ferrer et al. recently performed a retrospective study on 61 SCLC-transformed cases, either with *EGFR* mutation or not (22). In this study, overall survival (OS) from the initial diagnosis was lower in the *EGFR-*mutated group compared with the non-*EGFR*-mutant group; however, OS from the time of transformation into SCLC was comparable between the two groups (22).

The early introduction of platinum–etoposide chemotherapy along with osimertinib may act as an effective therapeutic strategy to eradicate emerging SCLC subclones and prevent the phenotypic transformation in *EGFR*-mutated patients with a high risk of SCLC switch (ongoing clinical trial, Clinicaltrials.gov: NCT03567642). Other approaches that might be pursued involve targeting cell cycle vulnerabilities generated upon *RB1* loss through the use of Aurora kinase (AURKA or AURKB) inhibitors (28, 29) or applying epigenetic therapy, mainly directly against reprogramming factors such as *EZH2* (30).

## Discussion

In the present study, we reported a case of osimertinib resistance driven by SCLC switch in an *EGFR*-mutated NSCLC patient. At the time of progression to osimertinib, due to phenotypic transformation, our patient received standard platinum–etoposide chemotherapy, achieving only modest clinical benefit.

Overall, it could be difficult to determine whether SCLC arises by transformation from NSCLC, rather than being a new tumor or being present simultaneously with the NSCLC from the beginning. Since SCLC is characterized by rapid growth and is not controlled by EGFR-TKIs, a simultaneous SCLC–NSCLC mixed tumor is expected to recur quickly. Our patient benefited from ~2 years of EGFR-TKIs; hence, it is unlikely that SCLC was part of the initial presentation.

A fundamental issue is represented by the identification of biomarkers able to predict SCLC transformation. Current evidence supports *TP53* and *RB1* mutations as potential predictors of phenotypic switch in *EGFR*-mutated NSCLC (23). In our case, histological examination at diagnosis showed the complete absence of p53 and Rb at IHC, likely underlying *TP53* and *RB1* baseline alterations. In addition, *TP53* mutations were detected by tissue NGS analysis before starting osimertinib, suggesting that the patient had a high risk of developing SCLC as a resistance mechanism.

To date, platinum–etoposide chemotherapy is the only viable treatment approach with a confirmed clinical efficacy in counteracting SCLC after failure of EGFR-TKIs. Given the positive results of the exploratory analysis of the IMpower150 trial in *EGFR*-mutated patients (31), a combination strategy of carboplatin–paclitaxel plus atezolizumab and bevacizumab after failure of previous EGFR-TKIs could be envisaged in this peculiar subset of patients, considering the proven efficacy of chemotherapy plus atezolizumab for the frontline treatment of extensive stage SCLC (32). Whether continued EGFR-TKI might gain additional clinical benefit is still a matter of debate, considering that SCLC is generally resistant to EGFR inhibition. Against this notion, a recent study reported a successful treatment with osimertinib in a synchronous SCLC and adenocarcinoma histology (33).

The continuation of osimertinib and its potential association with chemotherapy is still under investigation (34), also in view of the results obtained from the phase III IMPRESS study that did not demonstrate any PFS or OS improvement by continuing gefitinib vs. placebo in combination with second-line, platinum-based chemotherapy in *EGFR*-mutated NSCLC (35). In our report, drug screening assay on primary cell cultures from post-osimertinib biopsy showed increased sensitivity to the combination of osimertinib with chemotherapeutic drugs compared with control (*p* < 0.5), suggesting a potential effective therapeutic option. Moreover, it is likely that the progression to platinum–etoposide was driven by the *EGFR*-positive component in our case. To support this, the liver biopsy performed after chemotherapy (LB3) showed pure adenocarcinoma histology, and the liquid biopsy revealed the restoration of *EGFR* T90M. In this view, we assume that the interruption of EGFR pressure might have unleashed *EGFR*-positive clones resulting in inexorable treatment failure. Considering that our patient experienced CNS disease progression to platinum–etoposide, the excellent CNS penetration of osimertinib and its effectiveness on brain metastases might lead to continue osimertinib along with platinum-based chemotherapy in this occurrence.

## Data Availability Statement

The datasets presented in this study can be found in online repositories. The names of the repository/repositories and accession number(s) can be found here: NCBI BioProject, Accession No: PRJNA698448.

## Ethics Statement

Ethical review and approval was not required for the study on human participants in accordance with the local legislation and institutional requirements. The patients/participants provided their written informed consent to participate in this study. Written informed consent was obtained from the individual(s) for the publication of any potentially identifiable images or data included in this article.

## Author Contributions

AL, RM, and MT designed the manuscript. AL and GM collected the clinical data and performed the literature review. RM conducted the molecular analyses. LG and NC conducted the IHC analyses. SL and RA isolated tumor cells from biopsy tissue and performed the drug screening assay. AO performed the liver biopsies. LV performed the thoracic surgery. All the authors contributed to the writing of the manuscript and approved the submitted version.

## Conflict of Interest

MT has been on advisory boards and received speakers' fees for AstraZeneca, Pfizer, Eli-Lilly, BMS, Novartis, Roche, MSD, Boehringer Ingelheim, Otsuka, Takeda, and Pierre Fabre. MT has received research grants from AstraZeneca and Boehringer Ingelheim. AL received speakers' fees for AstraZeneca. The remaining authors declare that the research was conducted in the absence of any commercial or financial relationships that could be construed as a potential conflict of interest.
